# Structural, electronic, magnetic, half-metallic, mechanical, and thermodynamic properties of the quaternary Heusler compound FeCrRuSi: A first-principles study

**DOI:** 10.1038/s41598-017-16324-2

**Published:** 2017-11-23

**Authors:** Xiaotian Wang, Houari Khachai, Rabah Khenata, Hongkuan Yuan, Liying Wang, Wenhong Wang, Abdelmadjid Bouhemadou, Liyu Hao, Xuefang Dai, Ruikang Guo, Guodong Liu, Zhenxiang Cheng

**Affiliations:** 1 School of Physical Science and Technology, Southwest University, Chongqing, 400715 P.R. China; 2 Institute for Superconducting &Electronic Materials (ISEM), University of Wollongong, Wollongong, 2500 Australia; 3 Laboratoire d’Étude des Matériaux & Instrumentations Optiques; Département Matériaux & Développement Durable; Faculté des Sciences Exactes; Université Djillali Liabès de Sidi Bel Abbès, Sidi Bel Abbès, 22000 Algeria; 4 Laboratoire de Physique Quantique, de la Matière et de la Modélisation Mathématique (LPQ3M), Université de Mascara, Mascara, 29000 Algeria; 5 School of Material Sciences and Engineering, Hebei University of Technology, Tianjin, 300130 P.R. China; 6 Beijing National Laboratory for Condensed Matter Physics, Institute of Physics, Chinese Academy of Sciences, Beijing, 100190 P.R. China; 7 Laboratory for Developing New Materials and their Characterization, University of Setif 1, Setif, 19000 Algeria

## Abstract

In this paper, we have investigated the structural, electronic, magnetic, half-metallic, mechanical, and thermodynamic properties of the equiatomic quaternary Heusler (EQH) compound FeCrRuSi using the density functional theory (DFT) and the quasi-harmonic Debye model. Our results reveal that FeCrRuSi is a half-metallic material (HMM) with a total magnetic moment of 2.0 μ_B_ in agreement with the well-known Slater-Pauling rule M_t_ = Z_t_ − 24. Furthermore, the origin of the half-metallic band gap in FeCrRuSi is well studied through a schematic diagram of the possible *d-d* hybridization between Fe, Cr and Ru elements. The half-metallic behavior of FeCrRuSi can be maintained in a relatively wide range of variations of the lattice constant (5.5–5.8 Å) under uniform strain and the c/a ratio (0.96–1.05) under tetragonal distortion. The calculated phonon dispersion, cohesive and formation energies, and mechanical properties reveal that FeCrRuSi is stable with an EQH structure. Importantly, the compound of interest has been prepared and is found to exist in an EQH type structure with the presence of some B2 disorder. Moreover, the thermodynamic properties, such as the thermal expansion coefficient α, the heat capacity C_V_, the Grüneisen constant γ, and the Debye temperature Θ_D_ are calculated.

## Introduction

In the field of magnetic materials, the topic of research about spintronics^1^ has undoubtedly become of great concern. Furthermore, half-metallic materials (HMMs)^2^ are attracting great attention recently because these type of materials provide novel functionalities in spintronic and magneto-electronic devices. The electronic structure of the HMMs is metallic in only one of the two spin channels, resulting in a 100% spin polarization of the electrons near the Fermi level. Among the HMMs based on different structures, the Heusler^3–5^ ones have a special importance because of their interesting physical properties, such as their high Curie temperatures and tunable electronic structure.

Several investigations^6–16^ on the HMMs based on equiatomic quaternary Heusler structure (EQH) (LiMgPdSn/Y structure, space group F-43m, #216), have been done. Compared with the pseudo ternary Heusler HMMs, the EQH ones have the advantage of lower power dissipation due to the lesser amount of disorder that exists in them^17^. In addition, Heusler-type thin films usually lose their predicted ultra-high spin polarization due to the appearance of disorder. The half-metallic properties of the EQH compounds are quite robust, however, against interfering effects^18^. Here, we have simply reviewed the studies of the EQH compounds as follows: First, some EQH compounds XYMZ, where X, Y, and M denote the 3d transition-metal-elements, such as CoFeMnZ (Z = Al, Ga, Si, Ge)^7^, CoFeCrZ (Z = Al, Ga, Ge)^10^, and CoMnCrAl^19^, have been predicted experimentally and/or theoretically to be novel HMMs. Then, the scope of the EQH based HMMs has been extended to the compounds including 4d transition-metal-elements or rare-earth-elements, such as CoRuFeZ (Z = Al, Ga)^20^, ZrCoTiZ (Z = Al, Ga, Si, and Ge)^21^, ZrFeVZ (Z = Al, Ga, In)^22^, YCoTiZ (Z = Si, Ge)^23^ and YCoCrZ (Z = Si, Ge, Ga, Al)^24^. The half-metallic/spin-flipping band gap values of these compounds are normally larger than those of the EQH compounds containing only 3d-transition-elements, which is beneficial to the stability of the half-metallicity in practical applications. Very recently, our work^25^ demonstrates that the EQH compound LuCoCrGe can become a highly dispersive (near-linear-dispersive) zero-gap HMM at its strained lattice constant. Motivated by above-mentioned information, we must point out that the 4d-transition-elements-contained HMMs seem to be monumental treasures and worth mining.

In 2006, Mizutani *et al*.^26^, via first-principle calculations, have investigated the HM properties and the stability of the ferromagnetic state in the (Fe_x_Ru_1−x_)_2_CrSi (0 ≤ x ≤ 1). In 2007 and 2009, the peculiar magnetic, structural, magnetotransport and electrical behaviors of Ru_2−x_Fe_x_CrSi have been reported experimentally by Hiroi *et al*.^27,28^. In current study, we mainly focus on the interesting physical properties of the 4d-transition-elements-contained EQH based HMM FeCrRuSi. The structural, electronic, magnetic, half-metallic, mechanical, and thermodynamic properties of the new EQH compound FeCrRuSi are studied using first-principles calculations in combination with the quasi-harmonic Debye model. The effects of the uniform strain and the tetragonal distortion on the half-metallic behaviors have been also discussed. Importantly, the phase stability of this new compound has been also studied experimentally. Our current work is likely to inspire consideration of the 4d-transition-elements-contained EQH  based HMMs for application in future spintronic devices.

## Results and Discussion

### Electronic, magnetic, and half-metallic behaviors

**Figure 1:**
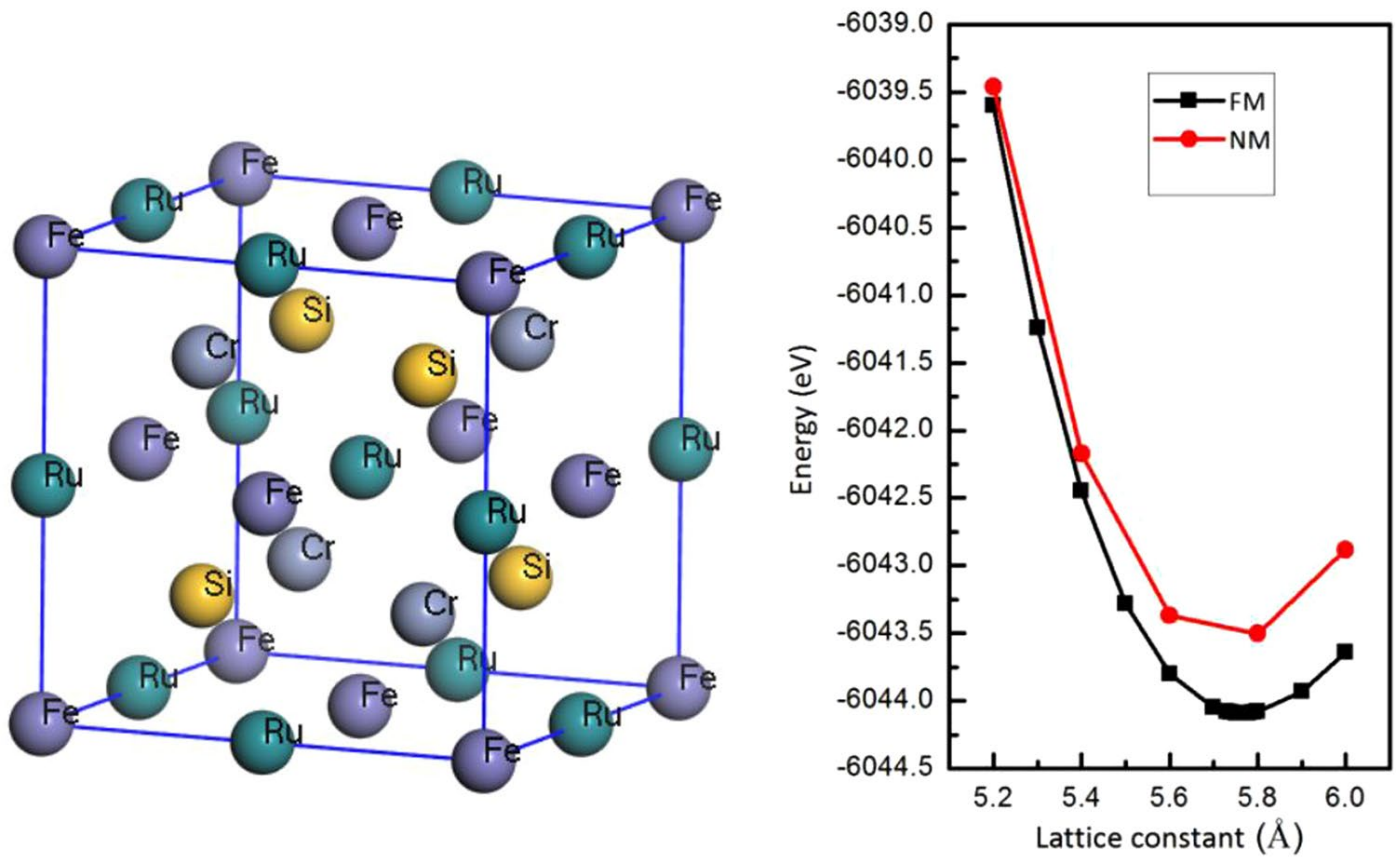
Crystal structure of EQH compound FeCrRuSi (left) and calculated total energies of FeCrRuSi compound with respect to the lattice constant. The NM (non magnetic) and FM (Ferromagnetc) states have been taken into account.

The Fe_2_CrSi compound has been synthesized and its physical properties were investigated by Luo *et al*.^29^. It is found that the L2_1_ structure is energetically more favorable than the XA structure. The Fe_2_CrSi compound exhibits half-metallic properties. The Ruthenium and Ferrum are in the same group of elements and have the same outermost valence electrons. When we use the Ruthenium to replace one of the Ferrum, a new EQH compound, FeCrRuSi, is achieved, as shown in Fig. 1. For the EQH compound FeCrRuSi, the Fe, Cr, Ru and Si atoms occupy the (0, 0, 0), (0.25, 0.25, 0.25), (0.5, 0.5, 0.5) and (0.75, 0.75, 0.75) Wyckoff positions, respectively.

**Figure 2:**
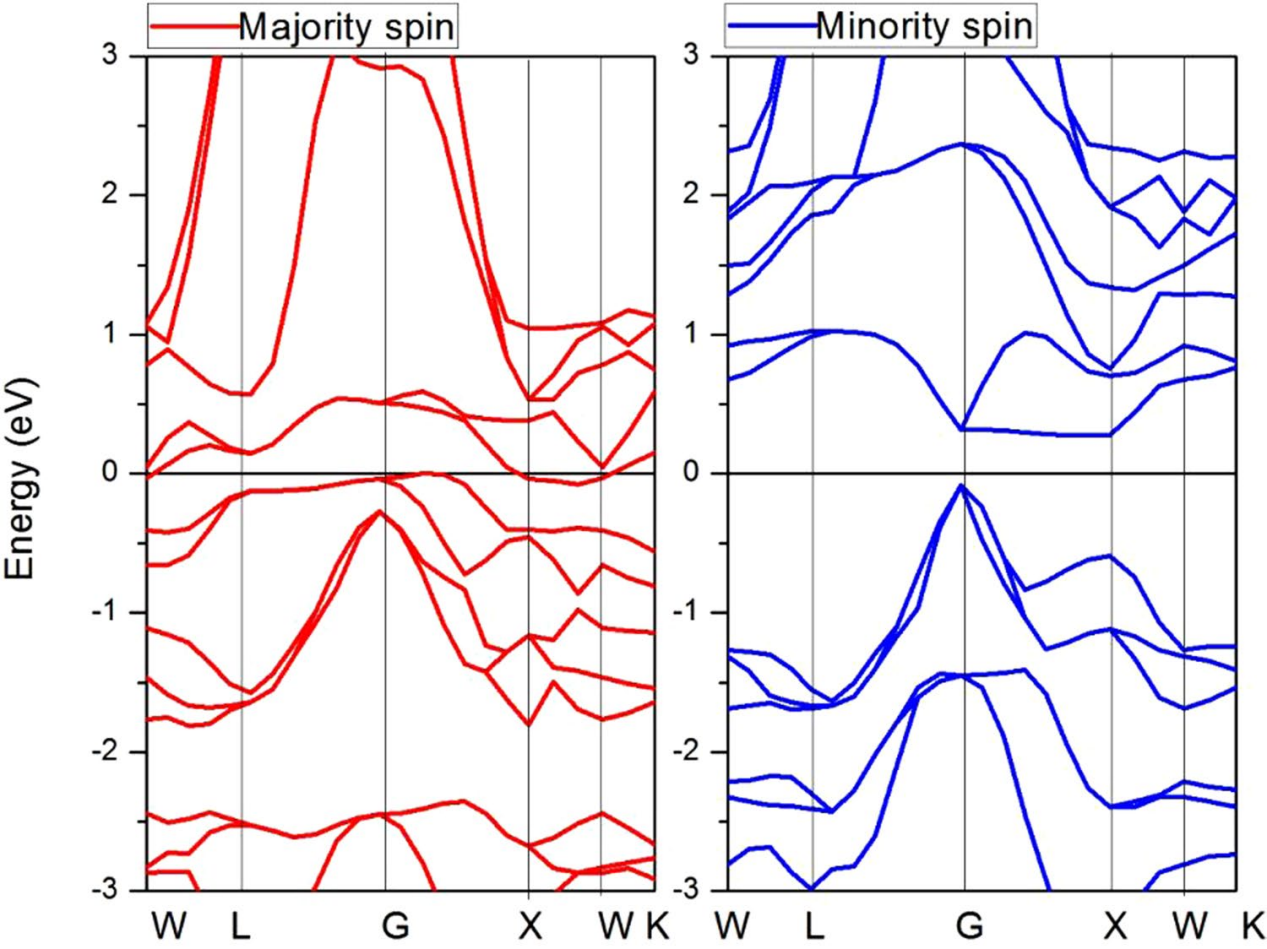
Calculated band structures of FeCrRuSi compound at its equilibrium lattice constant.

In order to determine the ground state properties of the FeCrRuSi compound, we perform a geometry optimization by calculating the total energy per unit cell at several lattice constants in both the ferromagnetic (FM) and nonmagnetic (NM) structures. Obviously, the total energy of the FM state is lower than that of the NM one, and the obtained equilibrium lattice constant in the FM state is 5.76 Å (see Fig. 1 and Table 1). Based on the equilibrium lattice constant, the electronic band structure of the FeCrRuSi compound has been calculated and displayed in Fig. 2. The Fermi level is located in the band gap in the minority spin channel. The valence band maximum (VBM) occurs at the Γ point in the Brillouin zone and the conduction band minimum (CBM) occurs at the X point in the Brillouin zone. The value of the indirect band gap is 0.384 eV in the minority spin channel. However, the majority spin band structure exhibits a metallic behavior. Hence, the EQH compound FeCrRuSi is a HMM.

**Table 1:** Calculated equilibrium lattice constant, total and individual atomic magnetic moments (μ_B_), number of valence electrons, spin polarization and possible Slater-Pauling (S-P) rule for the EQH compound FeCrRuSi.

Compound	Total	Fe	Cr	Ru	Si	a (Å)	Z_t_	S-P rule	P (%)
FeCrRuSi	2.00	−0.37	2.82	−0.44	−0.01	5.76	26	M_t_ = Z_t_−24	100

### Origin of the half-metallic band gap and the Slater-Pauling rule

**Figure 3:**
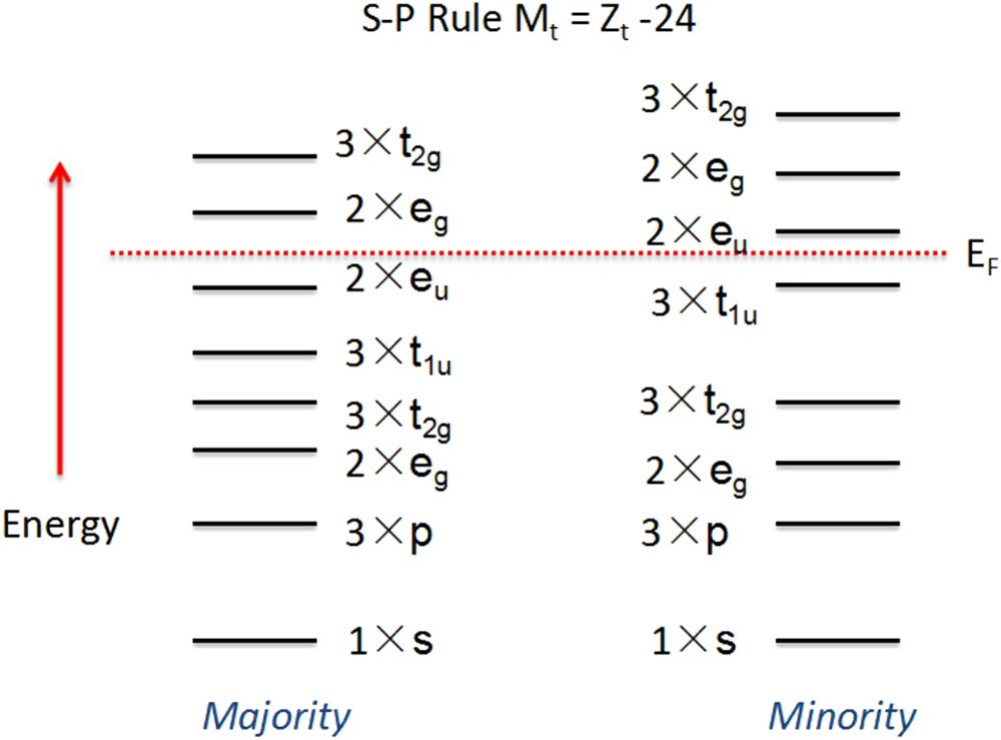
Schematic representation of the band structure for the FeCrRuSi EQH compound.

To further analyze the origin of the band gap in the minority spin channel, we show a schematic diagram of the energy levels of the spin-up (majority-spin) and spin-down (minority-spin) band structures for FeCrRuSi in Fig. 3. In view of Fig. 3, one can see that the double degeneracy *e*_u_ states are not occupied in the spin-down channel, and therefore, a *e*_u_ (non-bonding) - *t*_1u_ (bonding) energy band gap is formed in the spin-down direction for this compound. Based on the generalized electron-filling rule^30^, for FeCrRuSi, the total number of occupied states is 14 and 12 in the spin-up and spin-down channels, respectively, and therefore, there is a total spin magnetic moment of 2μ_B_. This theory is found to be in line with our calculated results, as shown in Table 1. Moreover, the total magnetic moment (M_t_) of FeCrRuSi is an integer value, which is a typical characteristic of the EQH compounds^25,27^. The EQH compound FeCrRuSi has 26 valence electrons (Z_t_) in its equilibrium lattice, obeying the Slater-Pauling rule^31^, M_t_ = Z_t_ −24. The atomic magnetic moments of the FeCrRuSi compound at its equilibrium lattice constant are also collected in Table 1. Clearly, the main contribution to the total magnetic moment comes from the Cr atoms, while the Fe and Ru atoms carry a part of the magnetic moments aligned anti-parallel to those of Cr atoms. This implies that the FeCrRuSi compound is an excellent half-metallic ferrimagnet at its equilibrium lattice constant.

**Figure 4:**
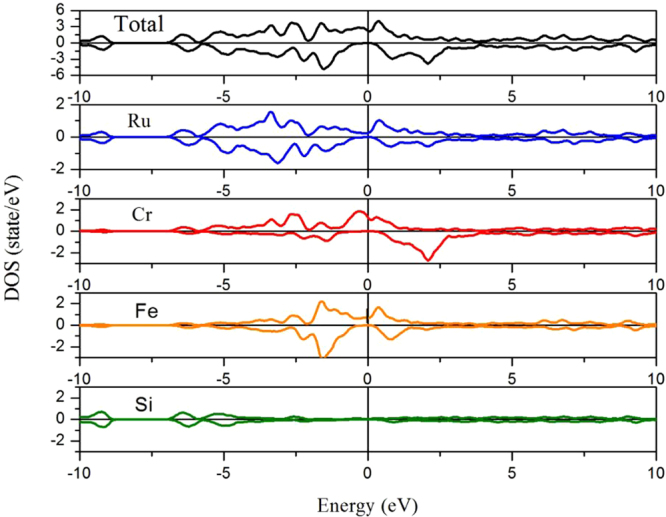
Calculated total and partial densities of states of FeCrRuSi EQH compound.

We further show in Fig. 4 the calculated total and partial densities of states (TDOS and PDOS) for the EQH compound FeCrRuSi at its equilibrium lattice constant. In the minority spin channel, the bonding states of the Fe atoms mainly located in the energy around −2 eV, whereas the antibonding states of the Cr atoms mainly sited in the energy near 2.5 eV, and therefore, the corresponding bonding-antibonding states led to the formation of an energy band gap. The spin polarization (P) of the FeCrRuSi compound at the Fermi level has been calculated using the following formula:

1 $${\rm{P}}=\frac{|N\uparrow ({E}_{f})-N\downarrow ({E}_{f})|}{|N\uparrow ({E}_{f})+N\downarrow ({E}_{f})|},$$

where $N\uparrow ({E}_{f})$ and $N\downarrow ({E}_{f})$ are the number of spin-up and spin down states, respectively. Based on the total DOS in Fig. 4, we find that the P of FeCrRuSi is 100%, reflecting that this compound could be useful for spin injection.

### Effect of the strain on the magnetic and half-metallic properties

**Figure 5:**
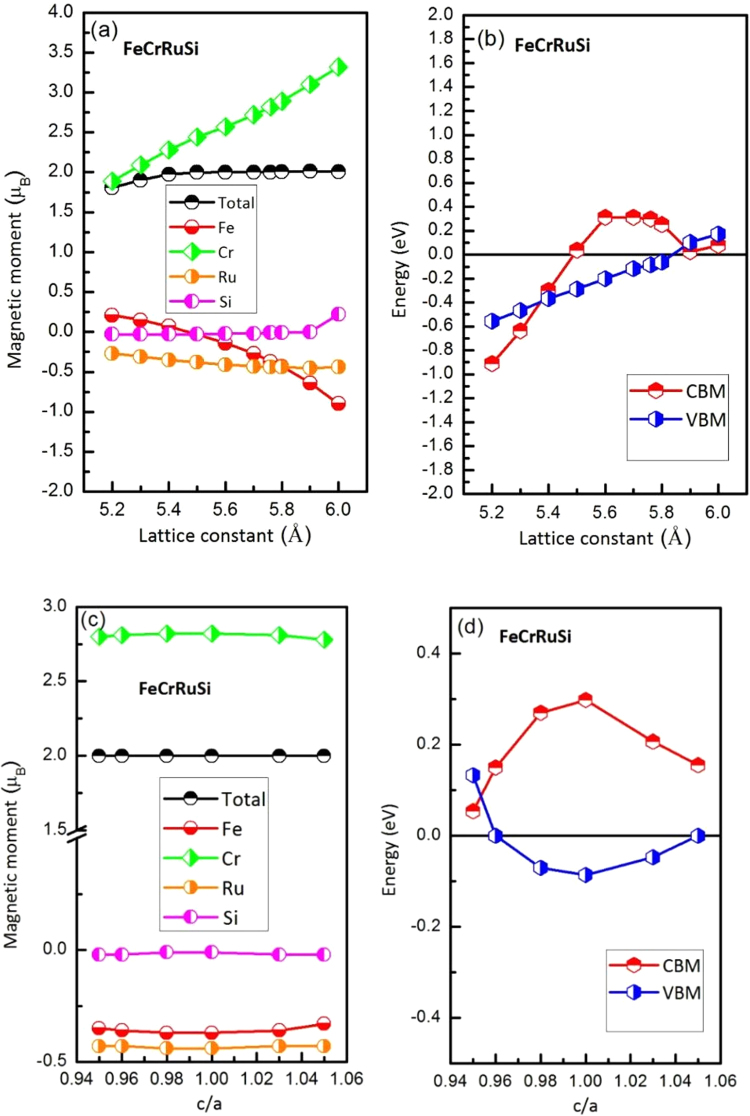
(**a**) Calculated total and atomic spin magnetic moments of FeCrRuSi as functions of the lattice constant; (**b**) dependence of the half-metallic states on the lattice constant (uniform strain); (**c**) calculated total and atomic spin magnetic moments of FeCrRuSi as functions of the c/a ratio; (**d**) dependence of the half-metallic states on the c/a ratio (tetragonal strain).

The total and atomic magnetic moments of the FeCrRuSi compound at its strained lattice constant are given in Fig. 5(a). The findings demonstrate the variation of the partial magnetic moment with respect to the contraction and the expansion of the lattice constant between 5.50 and 5.80 Å. The total magnetic moment is always equal to the fixed integer value of 2 µ_B_ at all the lattice constant values mentioned above. The magnetic moment values for the Fe and Ru atoms decrease with increasing lattice constant, whereas for the Cr atom, it continuously increases. Furthermore, in order to examine the robustness of the half-metallicity with respect to the change of lattice constant, the electronic structures of FeCrRuSi at different lattice constants (from 5.2 Å to 6.0 Å) are calculated. In this discussion, the values of the CBM and VBM for the FeCrRuSi compound in the minority spin channel have been recorded to show the half-metallic behavior for clarity, as shown in Fig. 5(b). When the value of the CBM is a positive number, and the value of the VBM is a negative number, FeCrRuSi is a HMM. But beyond that, the half-metallic behavior and the 100% spin polarization of FeCrRuSi are destroyed. From Fig. 5(b), we can observe that the half-metallic states of the EQH compound FeCrRuSi can be kept in the lattice constant value range of 5.50~5.80 Å. Also, the effect of a tetragonal distortion by varying the c/a ratio, with conserving the unit-cell volume, on the magnetic moments and the half-metallic properties have been investigated, as shown in Fig. 5(c) and (d). It is clear that the total and atomic magnetic moments of FeCrRuSi are nearly unchanged and the half-metallic states can be kept in the c/a ratio range of 0.96~1.05.

### Thermodynamic properties

To get more information about the specific behavior of a material when it is under severe constraints, e.g. high pressure and high-temperature environments, it is important to understand its thermodynamic properties. Hence, it become necessary to investigate the effects of pressure and temperature on thermodynamic parameters, such as thermal expansion coefficient α, heat capacity C_V_, Grüneisen constant γ, and Debye temperature Θ_D_. Here, we applied the quasi-harmonic Debye model^32^ to investigate the thermodynamic properties of the FeCrRuSi compound. The thermal properties are determined in the temperature range from 0 to 1200 K at some fixed pressures in the 0–45 GPa range.

In the quasi-harmonic Debye model^32^, the non-equilibrium Gibbs free energy of a solid is given by the following expression:

2 $${G}^{\ast }(V;P,T)=E(V)+PV+{A}_{{\rm{Vib}}}({\theta }_{D}(V);T)$$

where *E*(*V*) is the total energy per unit cell of the material, ${\theta }_{D}(V)$ is the Debye temperature and *A*_Vib_ is the vibrational Helmholtz free energy, which is defined as follows:

3 $${A}_{{\rm{Vib}}}({\theta }_{D};T)=nkT[\frac{9}{8}\frac{{\theta }_{D}}{T}+3\,\mathrm{ln}(1-{e}^{-{\theta }_{D}/T})-D({\theta }_{D}/T)]$$

where *n* is the number of atoms per formula unit, $D(y)$ is the Debye integral defined as follows:

4 $$D(y)=\frac{3}{{y}^{3}}{\int }_{0}^{y}\frac{{x}^{3}}{{e}^{x}-1}{\rm{d}}x$$

The Debye temperature of an isotropic solid can be computed as:

5 $${\theta }_{D}=\frac{h}{k}{[6{\pi }^{2}{V}^{1/2}n]}^{1/3}f(\sigma )\sqrt{\frac{{B}_{s}}{M}}$$

where *M* is the molecular mass per formula unit, *B*_*S*_ the static bulk modulus, which is defined by the following expression:

6 $${B}_{S}={B}_{static}=V(\frac{{{\rm{d}}}^{2}E(V)}{{\rm{d}}{V}^{2}})$$

and $f(\sigma )$ is given as:

7 $$f(\sigma )={\{3{[2{(\frac{2}{3}\frac{1+\sigma }{1-2\sigma })}^{2/3}+{(\frac{1}{3}\frac{1+\sigma }{1-\sigma })}^{2/3}]}^{-1}\}}^{1/3}$$

where *σ* is the Poisson ratio.

The equilibrium volume $V(T,P)$ curve (equation of state (EOS)) can obtained from the equation:

8 $${(\frac{\partial {G}^{\ast }(V;P,T)}{\partial V})}_{P,T}=0$$

The isothermal bulk modulus *B*_*T*_ is defined as follows:

9 $${B}_{T}(T,P)=-V{(\frac{\partial P}{\partial V})}_{T}$$

where the derivative is computed at the equilibrium volume at *T* and *P*. *B*_T_ can be more conveniently expressed as:

10 $${B}_{T}(T,P)=V{(\frac{{\partial }^{2}{G}^{\ast }(V;P,T)}{\partial {V}^{2}})}_{P,T}$$

The process of minimization and derivation involved in Eqs (8) and (10) is described in ref.^32^.

The heat capacity *C*_*V*_ and *C*_*p*_ can calculated from the following expressions:

11 $${C}_{V,{\rm{vib}}}=3n{k}_{B}[4D({\theta }_{D}/T)-\frac{3{\theta }_{D}/T}{{e}^{{\theta }_{D}/T}-1}]$$

12 $${C}_{P,{\rm{vib}}}={C}_{V,{\rm{vib}}}(1+{\alpha }_{V}{\gamma }_{{\rm{th}}}T)$$

where *α*_*V*_ represent the volume thermal expansion and ${{\rm{\gamma }}}_{th}$ is the thermal Grüneisen parameter, which are defined as:

13 $${\alpha }_{V}=\frac{{\gamma }_{{\rm{th}}}{C}_{V,{\rm{vib}}}}{{B}_{T}V}\,{\rm{and}}\,{\gamma }_{{\rm{th}}}=-\frac{{\rm{d}}\,\mathrm{ln}\,{\theta }_{D}(V)}{{\rm{d}}\,\mathrm{ln}\,V}$$

**Figure 6:**
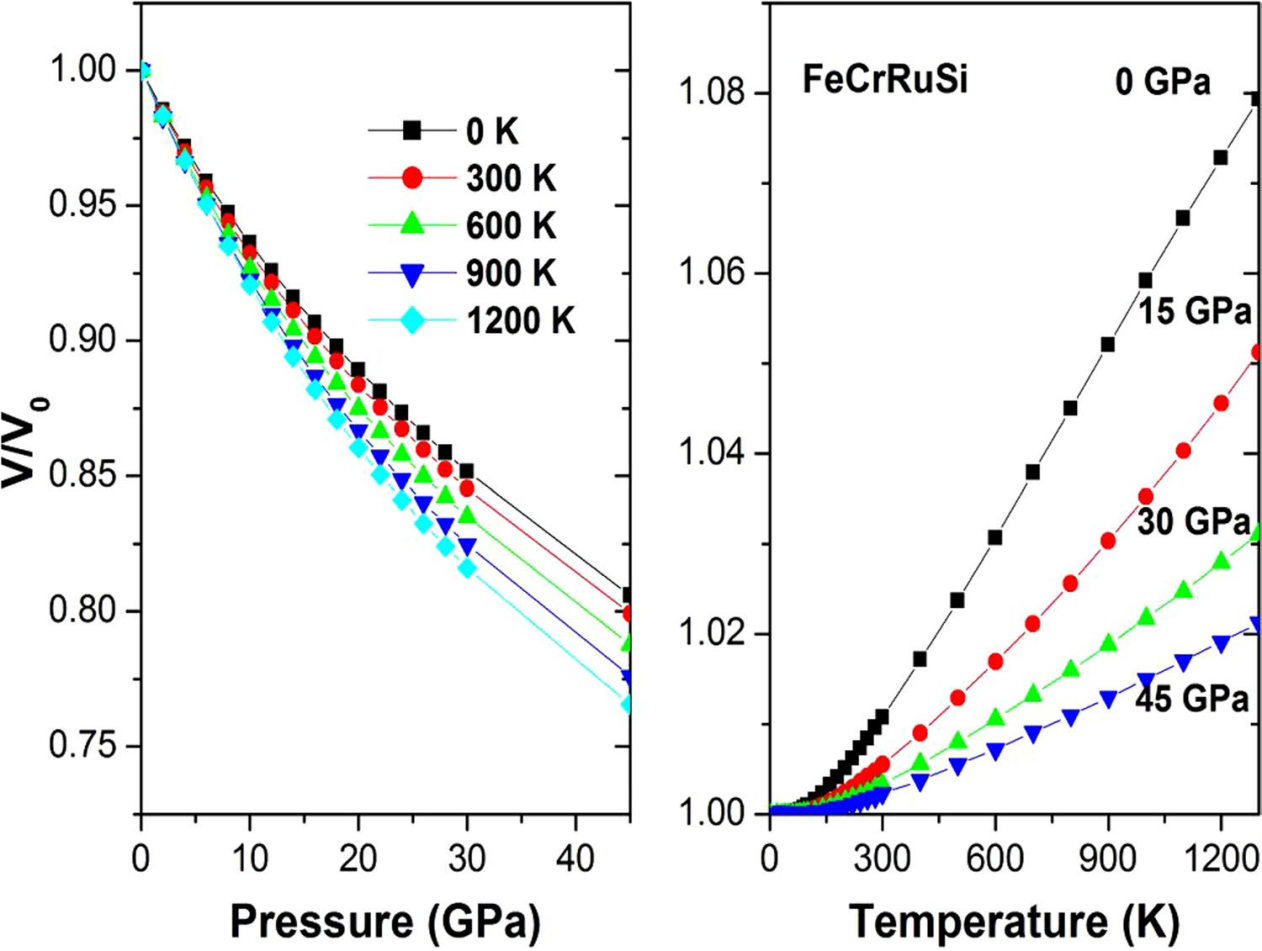
The normalized volume V/V_0_ versus (**a**) pressure and (**b**) temperature for FeCrRuSi.

Figure 6 shows the variation of the normalized primitive cell volume *V*/*V*_0_ versus temperature at some fixed pressures for FeCrRuSi, where *V* is the volume of the primitive cell at pressure *P* and *V*_0_ is its zero pressure equilibrium volume. The primitive cell volume increases with increasing temperature but the rate is more important for temperature range above 300 K. On the other side, as the pressure *P* increases, *V*/*V*_0_ decreases at a given temperature, and *V*/*V*_0_ at higher temperature is less than that at lower temperature at the same pressure.

**Figure 7:**
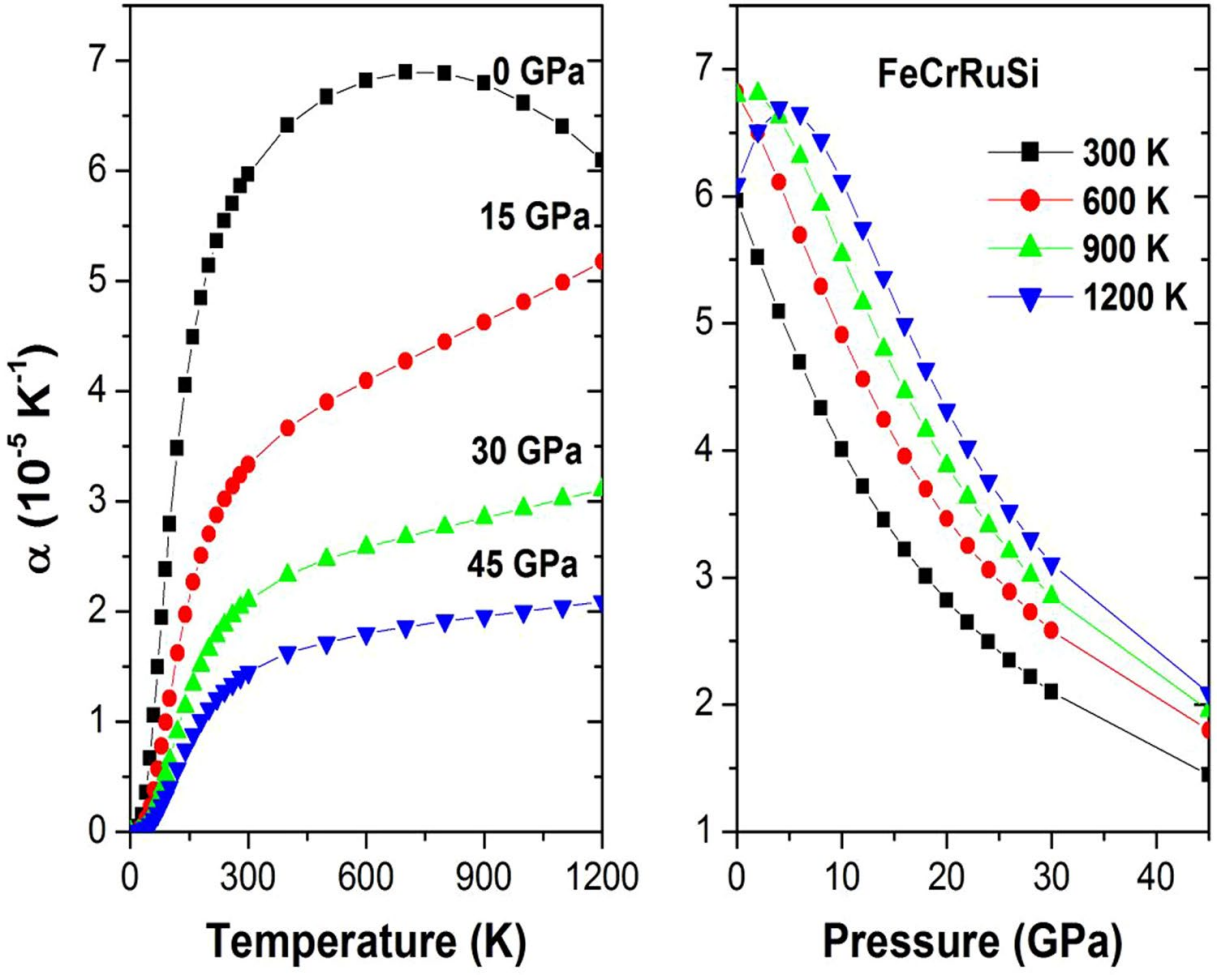
The thermal expansion coefficient α versus (**a**) pressure and (**b**) temperature for FeCrRuSi.

The thermal expansion coefficient *α* has an important theoretical and experimental significance and is also essential for predicting the thermodynamic equation of state. Figure 7 presents the effect of the temperature and pressure on the thermal expansion coefficient *α*. It is shown that *α* increases (decreases) with increasing temperature (pressure). For a given temperature, the thermal coefficient *α* decreases strongly with increasing pressure. For a given pressure, the thermal coefficient *α* increases sharply with increasing temperature up to 300 K. Above this temperature, *α* converges to a nearly constant value at high temperature. At zero pressure and 300 *K*, the thermal expansion *α* for the studied compound is 5.97 × 10^−5^ K^−1^.

**Figure 8:**
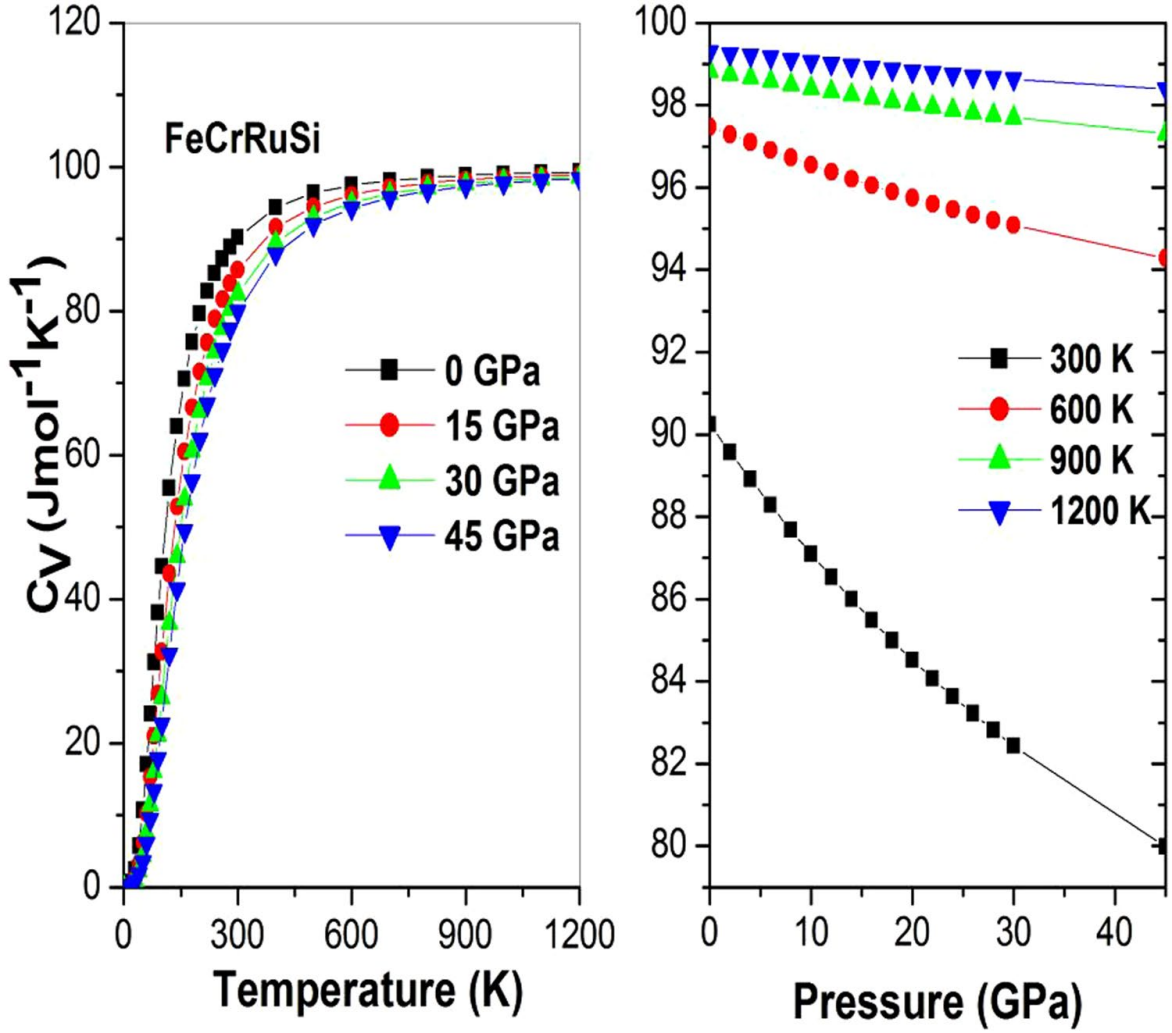
The heat capacity C_V_ versus (**a**) temperature and (**b**) pressure for FeCrRuSi.

The lattice vibration properties can be accessed through the heat capacity of a material. Therefore, the heat capacity at constant volume, *C*_*V*_, was calculated as a function of temperature at some fixed pressures, as shown in Fig. 8. Obviously, the *C*_*V*_ curve increases sharply up to 350 K, then it increases very slowly. At further high temperature *C*_*V*_ tends to approach the Dulong-Petit limit, indicating that the thermal energy at high temperature excites all the phonon modes, which is common to all solids at high temperature^33^. Figure 8 clearly indicates that at $T$ < 300 K, the heat capacity *C*_*V*_ depends on both temperature and pressure (*C*_*V*_ is proportional to *T* ^3^)^34^. From Fig. 8, one can note that the temperature and pressure have opposite influences on the heat capacity, and the effect of temperature on the heat capacity is more significant than that of the pressure. At high temperature *C*_*V*_ approaches approximately 99.51 JMol^−1^K^−1^. At zero pressure and 300 K, the calculated value of *C*_*V*_ is found to be equal to 89.78 JMol^−1^K^−1^.

**Figure 9:**
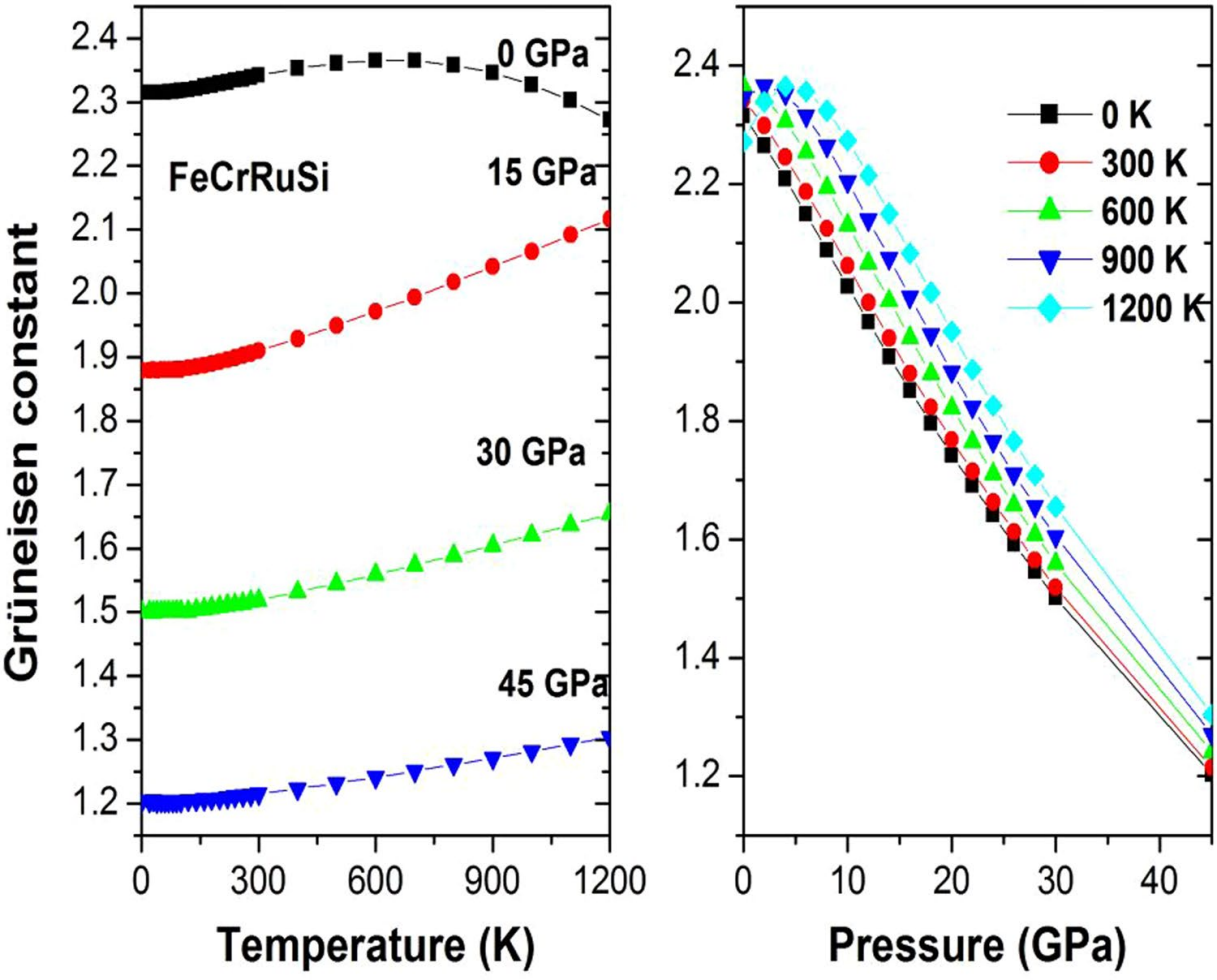
The Grüneisen constant γ versus (**a**) pressure and (**b**) temperature for FeCrRuSi.

The Grüneisen constant γ appears in some useful thermodynamic relations, therefore, it is significant to calculate it. Figure 9 shows the variation of the Grüneisen constant γ with temperature and pressure. It can be observed that γ is nearly constant from 0 K to 300 K, then γ increases linearly with increasing temperature. For a given temperature, γ decreases with pressure. The calculated γ of FeCrRuSi at room temperature and zero pressure is 2.353.

**Figure 10:**
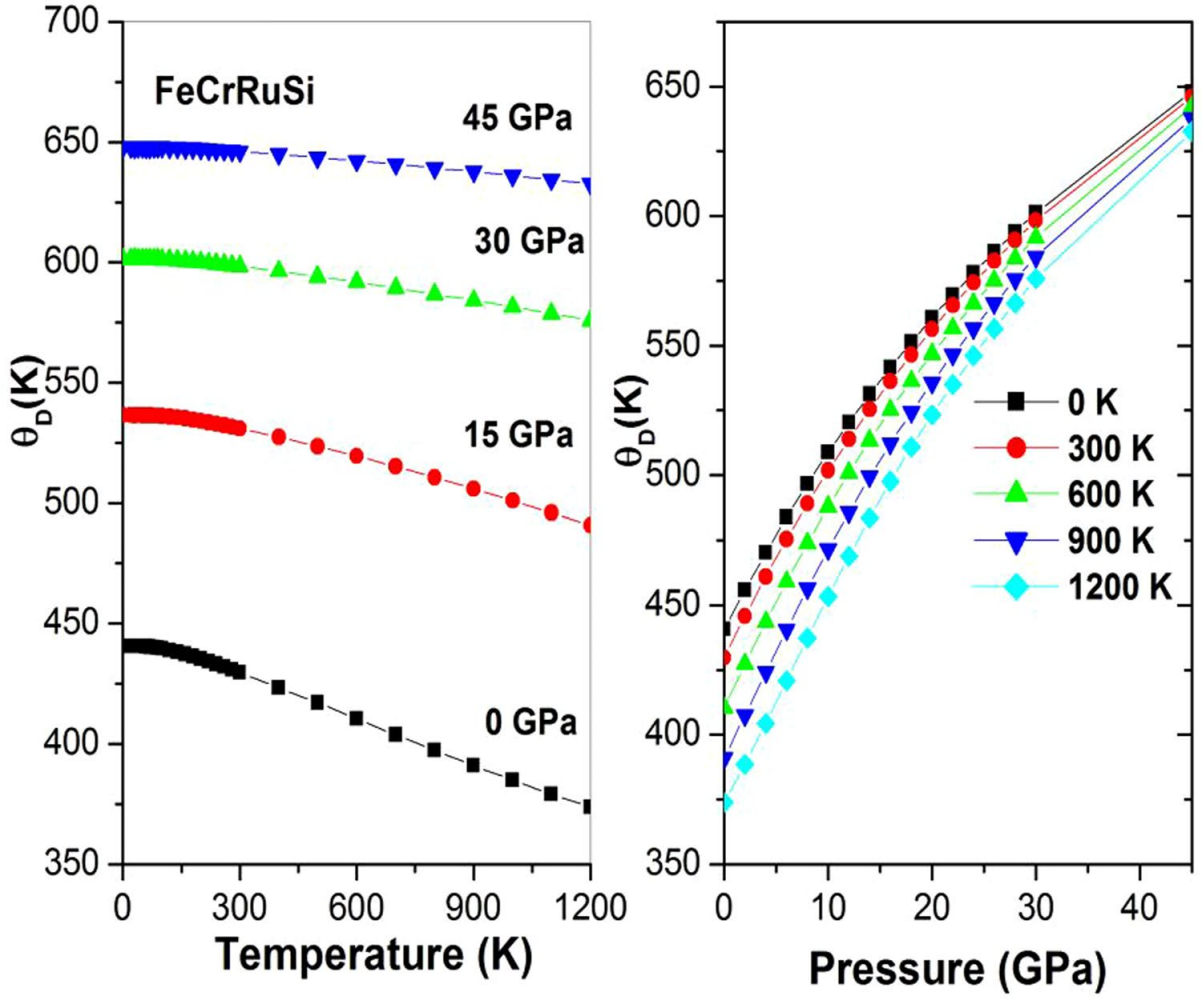
The Debye temperature *Θ* versus (**a**) pressure and (**b**) temperature for FeCrRuSi.

Finally, the evolution of Debye temperature *Θ* with temperature at some fixed pressures has been investigated, as shown in Fig. 10. It can be seen that *Θ* is nearly constant from 0 to 300 K and then decreases linearly with increasing temperature. For a given temperature, the Debye temperature increases with the enhancement of pressure. Our calculated *Θ* at zero pressure and ambient temperature is found to be equal to 435.14 K.

Up to now, there is no experimental data or theoretical results about the thermodynamic properties of the FeCrRuSi compound, so, our work is likely to provide a helpful reference for further investigations.

### Mechanical properties

In this section, we will focus on the mechanical behaviors of the FeCrRuSi compound. Cubic crystals have only three independent single-crystal elastic constants, namely, C_11_, C_12_, and C_44_. From the single-crystal elastic constants, one can calculate other important elastic moduli through the following equations^35^:

14 $$B=\frac{{C}_{11}+2{C}_{12}}{3}$$

15 $$G=\frac{{G}_{R}+{G}_{V}}{2}$$

16 $${G}_{V}=\frac{{C}_{11}-{C}_{12}+3{C}_{44}}{5}$$

17 $${G}_{R}=\frac{5({C}_{11}-{C}_{12}){C}_{44}}{4{C}_{44}+3({C}_{11}-{C}_{12})}$$

18 $$E=\frac{9GB}{3B+G}$$

19 $$A=\frac{2{C}_{44}}{{C}_{11}-{C}_{12}}$$

Here, *G* is the shear modulus, *B* is the bulk modulus, *G*_V_ is the Voigt’s shear modulus, *G*_R_ is the Reuss’s shear modulus, *E* is the Young’s modulus, and *A* is the anisotropy factor.

First, the mechanical stability of FeCrRuSi was examined according to the Born-Huang^36^ generalized elastic stability criteria:

20 $${C}_{44} > 0$$

21 $$\frac{({C}_{11}-{C}_{12})}{2} > 0$$

22 $$B > 0$$

23 $${C}_{12} > B > {C}_{11}$$

The calculated elastic constants (Table 2) verify the mechanical stability criteria. Hence, FeCrRuSi is mechanically stable. The *B*/*G* ratio is equal to 1.98, indicating that this compound is ductile based on the Pugh’s criteria^37^. Finally, the anisotropy factor (*A*) has been calculated to predict the anisotropic or isotropic behavior of FeCrRuSi. As shown in Table 2, the value of the anisotropy factor *A* deviates from the unity, indicating that FeCrRuSi is elastically anisotropic.

**Table 2:** Calculated elastic constants *C*_ij_, bulk modulus *B*, shear modulus *G*, Young’s modulus *E* (GPa), Pugh’s ratio *B*/*G*, anisotropy factor *A*, and formation and cohesive energies (eV) for the EQH compound FeCrRuSi.

EQH compound	C_11_	C_12_	C_44_	B	G	E	A	B/G	Formation energy	Cohesive energy
FeCrRuSi	361.1	181.9	141.6	241.7	121.9	239.1	1.60	1.98	−1.74	24.18

### Formation and cohesive energies, and phonon dispersion

In this section, the cohesive and formation energies have been calculated in order to check the structural stability of the FeCrRuSi compound. We should point out that similar analysis about the structural stabilities of Heusler compounds can be found in some references^38–41^. First, we calculate the cohesive energy via the formula:

24 $${E}_{coh}^{FeCrRuSi}=({E}_{Fe}+{E}_{Cr}+{E}_{Ru}+{E}_{Si})-{E}_{total}^{FeCrRuSi},$$

where E_Fe_, E_Cr_, E_Ru_, and E_Si_ are the isolated atomic energies of the Fe, Cr, Ru and Si atoms, respectively, and ${E}_{total}^{FeCrRuSi}$ is the total energy of FeCrRuSi per formula unit. The calculated cohesive energy is found to be equal to 24.18 eV which is very large (even larger than 20 eV), indicating the chemical stability of FeCrRuSi. The formation energy is calculated using the following expression:

25 $${E}_{f}^{FeCrRuSi}={E}_{FeCrRuSi}^{total}-({E}_{Fe}^{bulk}+{E}_{Cr}^{bulk}+{E}_{Ru}^{bulk}+{E}_{Si}^{bulk}),$$

where ${E}_{total}^{FeCrRuSi}$ is the total energy of FeCrRuSi per formula unit, and ${E}_{Fe}^{bulk}$, ${E}_{Cr}^{bulk}$,${E}_{Ru}^{bulk}$ and ${E}_{Si}^{bulk}$ are the total energies of the Fe, Cr, Ru and Si bulks, respectively. The calculated formation energy is equal to −1.74 eV, indicating the structural stability of the considered compound, and thus this compounds may be synthesized using conventional equilibrium methods such as arc-melting.

**Figure 11:**
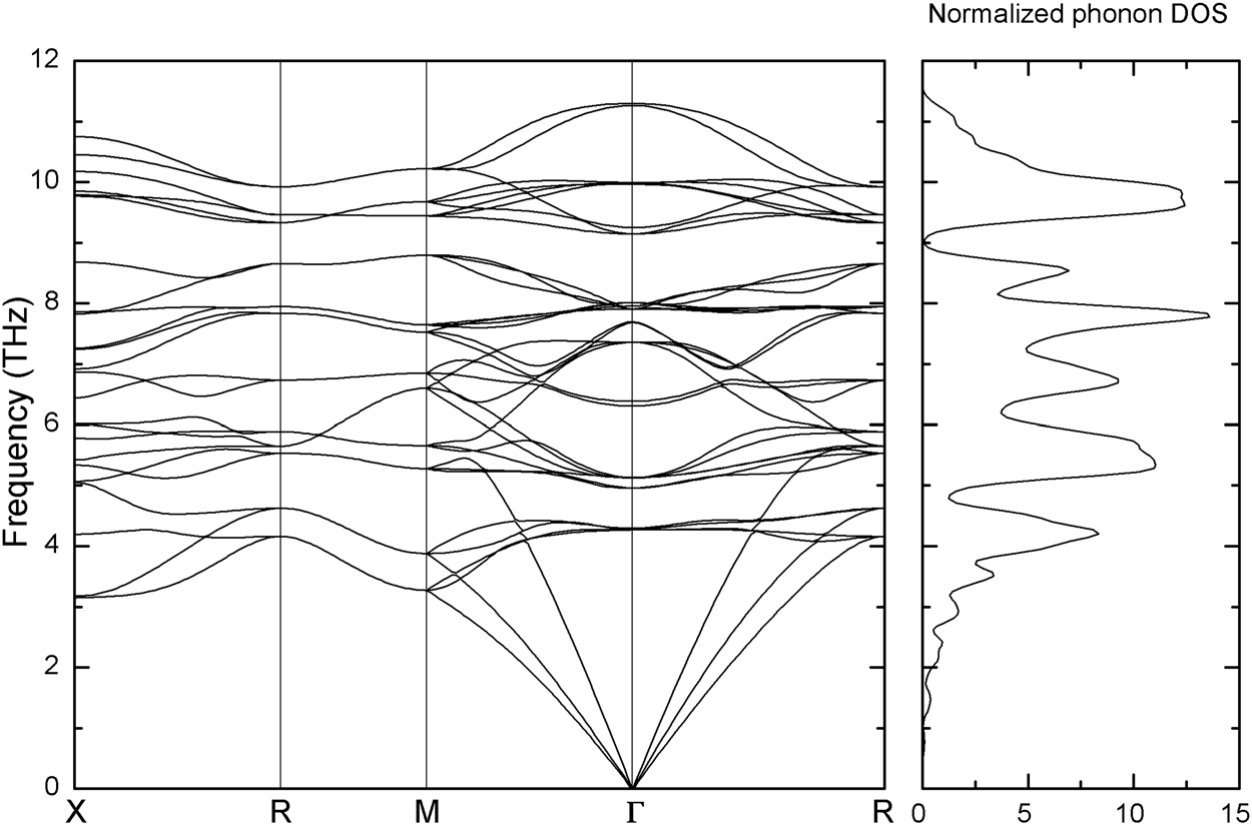
Calculated phonon dispersion curves and phonon DOS for FeCrRuSi at 0 GPa.

To further elucidate the dynamical stability of the FeCrRuSi compound, we have also calculated its phonon dispersion along the X-R-M-Γ-R directions in the Brillouin zone and the corresponding density of state (DOS) at its equilibrium lattice constant, which are displayed in Fig. 11. It is clearly seen that the phonon dispersion spectrum has no imaginary frequencies, indicating the dynamical stability of the FeCrRuSi compound.

### Structural properties in experiment

In this section, we will make an outlook about the measured structural properties. Fig. S1 shows the XRD patterns of the EQH compound FeCrRuSi annealed at 773 K for 3 days. The experimental lattice constant value of FeCrRuSi is equal to 5.758 Å, which is in agreement with our calculated equilibrium lattice constant (5.76 Å). Obviously, this compound is found to exist in the EQH type (labelled also Y-type) crystal structure, however, the superlattice reflections (111) and (200) are observed to be present in FeCrRuSi. Moreover, the intensity of (200) peak is much larger and can be observed clearly, while (100) is weak, reflecting an evidence of B2 disorder^42^. Noted that, Picozzi *et al*.^43^ reported that the half-metallic behaviors in Heusler compounds (Co_2_MnGe/Si) can be maintained in presence of B2 disorder. The magneto-transport measurement of the FeCrRuSi compound should be performed in the follow-up work.

## Summary

Using first-principles calculations and the quasi-harmonic Debye model, the structural, electronic, magnetic, half-metallic, mechanical, thermodynamic and possible Slater-Pauling behaviors of a newly designed EQH compound FeCrRuSi have been investigated in detail. Our calculation results indicate that the EQH compound FeCrRuSi is a HMM with a total magnetic moment of 2 μ_B_ and it follows the well-known Slater-Pauling rule M_t_ = Z_t_ − 24. Furthermore, the origin of the half-metallic band gap of FeCrRuSi is *e*_u_ (non-bonding) - *t*_1u_ (bonding) energy band gap in the spin-down direction. The half-metallic behavior of FeCrRuSi can be maintained for a relatively wide range of the lattice constant variations (5.5–5.8 Å) under a uniform strain and c/a ratio variations (0.96–1.05) under a tetragonal distortion, respectively. The quasi-harmonic Debye model is successfully applied to examine the thermodynamic behaviors of FeCrRuSi at different temperatures and pressures. FeCrRuSi is mechanically stable according to Born-Huang elastic stability criteria. FeCrRuSi exhibits ductile and anisotropic characters. The considered EQH compound is energetically stable according to the calculated cohesive and formation energies, and phonon dispersion. Importantly, the FeCrRuSi compound has been prepared. It exists in the EQH type structure with presence of B2 disorder. The present work suggests that the EQH FeCrRuSi compound is useful in spintronic applications.

## Method of Calculations

To investigate the structural, electronic and magnetic properties of the FeCrRuSi compound, we have performed first-principles calculations using the pseudo-potential plane-wave method^44^ as implemented in the Cambridge Serial Total Energy Package (CASTEP) code^45^. The CASTEP code is an effective ab initio program based on quantum mechanics. It can precisely simulate the ground structure, band structure, optical properties, magnetic properties, and so on. The interactions between the atomic core and the valence electrons were described by the ultrasoft pseudo-potential approach. The generalised gradient approximation (GGA)^46,47^ was adopted for the exchange-correlation potential. For all cases, a plane-wave basis set cut-off of 450 eV was used. A *k*-point mesh of 12 × 12 × 12 was used in the Brillouin zone integrations. These parameters ensured good convergence of the total energy. The convergence tolerance for the calculations was selected as a difference in the total energy within 1 × 10^−6^ eV/atom.

Furthermore, the thermodynamic properties of this compound are predicted through the quasi-harmonic Debye model, in which the lattice vibrations are taken into account. The variation of the relative volume, thermal expansion, heat capacity, Grüneisen parameters and the Debye temperature with pressure and temperature are successfully obtained.

For the phonon spectrum of FeCrRuSi, we have employed the finite displacement method as implemented in the Vienna *ab initio* simulation package (VASP)^48^ code based on the first-principles and the projector-augmented wave method (PAW)^49^ within the GGA-PBE. An energy cutoff of 500 eV and a 5 × 5 × 7 k-mesh in the Brillouin zone were adopted for the calculations of phonon spectrum.

It is worth to mention that the polycrystalline ingot of FeCrRuSi in this work was prepared by arc melting under a protective argon atmosphere. More details about the experimental procedure can found in the supplementary material.

## Electronic supplementary material

Supplementary Material
