# Supplementary material for: Structural, electronic, magnetic, half-metallic, mechanical, and thermodynamic properties of the quaternary Heusler compound FeCrRuSi: A first-principles study
Source: Sci Rep. 2017 Nov 23;7:16183. doi: 10.1038/s41598-017-16324-2 (PMC5701081; doi:10.1038/s41598-017-16324-2)
Supplement: Supplementary file 1 — Supplementary Material [file 41598_2017_16324_MOESM1_ESM.doc]

**Supplementary Material**

Structural, electronic, magnetic, half-metallic, mechanical, and thermodynamic properties of the quaternary Heusler compound FeCrRuSi: A first-principles study

Xiaotian Wang^a, b^, Houari Khachai^c^, Rabah Khenata^d^, Hongkuan Yuan^a^, Liying Wang^e^, Wenhong Wang^f^, Abdelmadjid Bouhemadou^g^, Liyu Hao^a^, Xuefang Dai^e^, Ruikang Guo^e^, Guodong Liu^e^, Zhenxiang Cheng^b, *^

*^a^ School of Physical Science and Technology, Southwest University, Chongqing 400715, PR China.*

*^b^Institute for Superconducting &Electronic Materials (ISEM), University of Wollongong, Wollongong 2500, Australia*

*^c^ Laboratoire d’Étude des Matériaux & Instrumentations Optiques; Département Matériaux & Développement Durable; Faculté des Sciences Exactes; Université Djillali Liabès de Sidi Bel Abbès 22000, Algeria.*

*^d^ Laboratoire de Physique Quantique, de la Matière et de la Modélisation Mathématique (LPQ3M), Université de Mascara, Mascara 29000, Algeria*

*^e^ School of Material Sciences and Engineering, Hebei University of Technology, Tianjin 300130, PR China*

*^f^ Beijing National Laboratory for Condensed Matter Physics, Institute of Physics, Chinese Academy of Sciences, Beijing 100190, PR China*

*^g^ Laboratory for Developing New Materials and their Characterization, University
of Setif 1, Setif 19000, Algeria*

*E-mail address:* [***cheng@uow.edu.au***](mailto:cheng@uow.edu.au) ***(Prof. Z. Cheng)****;*

**Expremental produce**

Polycrystalline ingot of FeCrRuSi was prepared by arc melting under a protective argon atomsphere and then annealed at 773 K for 3 days. Its structure was studied by powder X-ray diffraction (XRD) using Cu-Kα radiation.


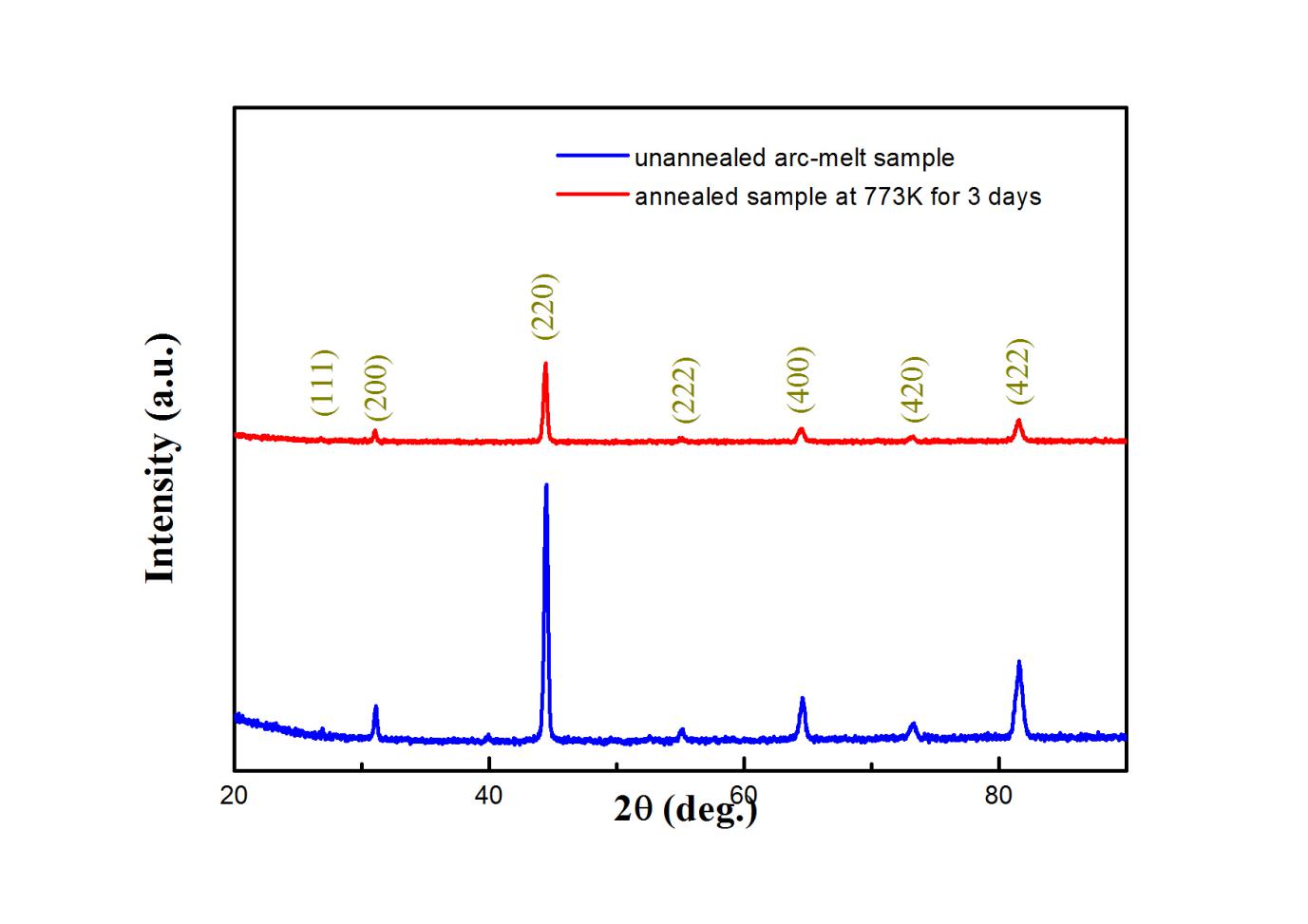


**Fig. S1** The powder XRD patterns for the arc-melt sample FeCrRuSi and the sample annealed at 773 K for 3 days.
